# Whole-Exome Sequencing of HPV Positive Tonsillar and Base of Tongue Squamous Cell Carcinomas Reveals a Global Mutational Pattern along with Relapse-Specific Somatic Variants

**DOI:** 10.3390/cancers14010077

**Published:** 2021-12-24

**Authors:** Andreas Ährlund-Richter, Stefan Holzhauser, Tina Dalianis, Anders Näsman, Michael Mints

**Affiliations:** 1 Department of Oncology-Pathology, Karolinska Institutet, Karolinska University Hospital, 171 64 Stockholm, Sweden; Andreas.Ahrlund@gmail.com (A.Ä.-R.); Stefan.Holzhauser@ki.se (S.H.); Tina.Dalianis@ki.se (T.D.); 2 Department of Clinical Pathology, CCK R8:02, Karolinska University Hospital, 171 64 Stockholm, Sweden; 3 Department of Medicine Solna, Karolinska Institutet, Karolinska University Hospital, 171 64 Stockholm, Sweden; 4 Department of Surgical and Perioperative Science, Urology and Andrology, Umeå University, 907 36 Umeå, Sweden; 5 Department of Molecular Cell Biology, Weizmann Institute of Science, Rehovot 76100, Israel

**Keywords:** human papillomavirus, HPV, oropharyngeal cancer, OPSCC, tonsillar cancer, base of tongue cancer, survival, recurrence, WES, targeted therapy, biomarkers

## Abstract

**Simple Summary:**

To better prevent/combat recurrence and identify predictive/targetable markers upon diagnosis, we performed whole-exome sequencing (WES) of primary tumours and relapses of human papillomavirus positive (HPV^+^) tonsillar and base of tongue cancer (TSCC/BOTSCC) on patients treated with curative intent, with and without relapse. A specific deletion in the *CDC27* gene was observed only in the primaries of 5/17 patients that recurred but in none of the 18 patients without recurrence. Furthermore, three specific variants and 26 mutated genes enriched in mucins were identified in at least 30% of all primaries irrespective of recurrence. To conclude, a specific *CDC27* deletion could be specific for recurrent HPV^+^ TSCC/BOTSCC, while *BCLAF1*, *AQP7* and other globally mutated genes could be of significance for further investigation.

**Abstract:**

To identify predictive/targetable markers in human papillomavirus positive (HPV^+^) tonsillar and base of tongue cancer (TSCC/BOTSCC), whole-exome sequencing (WES) of tumours of patients with/without recurrence was performed. Forty primary tumours and adjacent normal tissue were separated by micro-dissection from formalin-fixed paraffin-embedded tissue from patients treated with curative intent 2000–2014 at Karolinska University Hospital. Successful sequencing was obtained in primary tumours of 18 patients without and primaries of 17 with local or distant recurrence, as well as in 10 corresponding recurrences (i.e., five local relapses and five distant metastases) from these 17 patients. One variant—a high-impact deletion in the *CDC27* gene—was observed only in primaries of 5/17 patients that had a recurrence after full treatment but in none of those without recurrence. In addition, 3 variants and 26 mutated genes, including *CDC27*, *BCLAF1* and *AQP7*, were present in at least 30% of all primary tumours independent of prognosis. To conclude, a *CDC27* deletion was specific and found in ~30% of samples from patients with a local relapse/distant metastasis and could, therefore, potentially be a prospective marker to predict prognosis. Commonly mutated genes, such as *BCLAF1*, should be further studied in the context of targeted therapy.

## 1. Introduction

Human papillomavirus positive (HPV^+^) tonsillar and base of tongue squamous cell carcinoma (TSCC/BOTSCC) patients have a better prognosis compared to those with HPV-negative (HPV^−^) TSCC/BOTSCC and other head and neck squamous cell carcinoma (HNSCC), but 15–20% still relapse [1,2,3,4]. Since the incidences of HPV^+^ TSCC and BOTSCC, the two oropharyngeal squamous cell carcinoma (OPSCC) subsites, where HPV is mainly found, are rising epidemically in many Western countries, improved therapeutic options are crucial for these patients [5,6,7,8,9,10,11,12]. Today, HPV^+^ TSCC/BOTSCC patients are often offered induction or concomitant chemoradiotherapy, but this has still not improved the survival of those with poor prognosis when compared to the previously given radiotherapy and surgery highlighting the need for new therapeutic options [13,14]. For the majority of HPV^+^ patients that have a good prognosis, the main clinical challenge is how to de-escalate treatment to decrease side effects while maintaining survival [14].

To improve patient stratification and individual tailoring of therapy, efforts have been made to uncover novel prognostic markers in HPV^+^ TSCC/BOTSCC/OPSCC [14,15,16,17,18,19,20,21,22,23,24,25,26,27,28,29,30,31,32,33,34,35,36,37,38,39]. Stage, age, HPV16 E2 mRNA expression, high CD8^+^ tumour infiltrating lymphocyte (TIL) counts, and many other molecular markers have been proposed as predictive of prognosis, and models combining multiple markers have been shown to identify 20–56% of all patients with a >95% probability of 3-year disease-free survival [15,17,19,20,22,26,28,29,30,31,32,33,34,35,36,37,38,39]. However, more biomarkers are required to improve these models and accurately identify a larger proportion of patients with a likely excellent prognosis so that these can receive de-escalated treatments.

On the other side of the spectrum, there is a significant need for druggable markers that can be used in targeted therapy—as first-line treatment for patients with poor prognosis and as salvage treatment for patients with recurrent disease [18]. In breast and urothelial cancers, the development of targeted therapy has been guided by identifying commonly mutated genes [40,41]. Targeting phosphatidylinositol-4,5-bisphosphate 3-kinase catalytic subunit alpha (PIK3CA) in breast cancer and fibroblast growth factor 3 (FGFR3) in urothelial cancer was attempted in clinical trials, e.g., NCT04524000 and NCT03390504, and is now approved for metastatic disease in these cancer types.

Similar approaches, including phosphoinositide 3-kinase (PI3K) inhibitors, have also been initiated in HNSCC patients, including OPSCC, with recurrent disease (e.g., NCT01602315 and NCT02537223). Notably, in OPSCC, it is now well known that PIK3CA and FGFR3 are more frequently mutated in HPV^+^ tumours, while TP53 is more frequently mutated in HPV^−^ tumours [18,37]. Moreover, the presence of mutated PIK3CA or FGFR3 has been shown to have prognostic significance in a subset of cases [15,18,39].

Specifically, in HPV^+^ OPSCC, there are a number of global mutation studies trying to map the differences in mutational patterns between tumours of recurrent versus nonrecurrent patients [22,32]. However, results differ between studies, suggesting that further investigations are needed in order to confidently say which mutations should be focused on for diagnostic and therapeutic purposes.

To further contribute to the knowledge in the field and find potential predictive and targetable markers, we performed whole-exome sequencing (WES) of primary HPV^+^ TSCC/BOTSCC in patients without and with local or distant recurrence, all treated with curative intent. In addition, WES was performed on five local recurrences and five distant metastases from the above patients. We focused on identifying genes and variants that were uniquely mutated in each cohort, in addition to detecting genes commonly mutated in both groups, the latter being representative of a general HPV^+^ TSCC/BOTSCC mutational pattern.

## 2. Materials and Methods

### 2.1. Patients, Samples and Definition of HPV-Positive Status

Twenty HPV^+^ TSCC/BOTSCC (ICD-10 codes C09.0-9 and C01.9 resp.) patients with local or distant recurrent disease (patients with recurrence) and 20 stage and age-matched patients without recurrence, fully treated 2000–2014 at Karolinska University Hospital, with formalin-fixed paraffin-embedded (FFPE) samples from the patients’ primary tumours were initially included. In addition, FFPE material from 5 local recurrences and 5 distant metastases was available from the patients with recurrence.

Having an HPV^+^ TSCC/BOTSCC was defined as having a primary tumour that was both HPV DNA positive and overexpressed p16^INK4A^ (p16) [42]. Data on the presence of HPV DNA and p16 overexpression in patient samples and treatment and clinical outcome of these patients were derived from previous studies [7,10,28,43]. In those studies, DNA was extracted, and HPV DNA status was assayed by PCR-based methodology as described before [3,7,10,28,43]. Briefly, all samples were assayed using a multiplex bead-based assay for 27 different HPV types [7]. These HPV types were: HPV 6, 11, 16, 18, 26, 30, 31, 33, 35, 39, 42, 43, 44, 45,51, 52, 53, 56, 58, 59, 66, 67, 68, 69, 70, 73 and 82, and β-globin was included as a positive control for the presence of DNA, as described previously [7]. Only samples with a β-globin median fluorescent value (MFI) of ≥30 were considered to have sufficient DNA quality to be assessed for HPV status and were included in this study. As a positive control for HPV DNA, DNA from the HPV16 positive SiHa cell line was included in the PCR and MagPix analysis.

In the same studies, p16 expression was examined using the monoclonal antibody (mAb) clone JC8 (Santa Cruz Biotech, Santa Cruz, CA, USA) and having >70% positivity was defined as having a p16 positive sample [7,10,28,43].

This study was conducted according to ethical permissions 99-237, 2005/431-31/4, 2009/1278-31/2; 2017/1035-31/2 and 2018/870-32 from the Stockholm Regional Ethical Review Board.

### 2.2. Laser Microdissection and DNA Extraction

In order to enrich the tumour tissue and to also obtain normal tissue for more accurate mutation calling, FFPE samples from both the primary tumours and the available metastases were laser micro-dissected using a Leica LMD 7000 microscope (Leica Microsystems, Wetzlar, Germany) and the Laser Microdissection System (version 7.6.5684) [44]. This way, we estimated having obtained >90% tumour tissue and virtually 100% normal tissue in the separate samples before DNA was extracted. DNA extraction was performed as described previously [8,10,28,43].

### 2.3. Library Preparation and Whole-Exome Sequencing

From the DNA extracted above, an amount of 40–250 ng DNA from each sample was used for the library preparation with KAPA HyperPlus (Roche, Pleasanton, CA, USA) according to the instructions of the manufacturer with some modifications. More specifically, fragmentation was performed with 12.5 min incubation, and xGen Duplex Seq adapters (3–4 nt) unique molecular identifiers (UMI) at 0.6 mM (Integrated DNA Technologies, Coralville, IA, USA) were used for the ligation, and xGen Indexing primers (2 mM) with unique dual indices (Integrated DNA Technologies, Coralville, IA, USA) were used for PCR amplification (5–13 cycles depending on input amount of DNA). Target enrichment was performed in a multiplex fashion with a library amount of 375 ng (4-plex). The libraries were hybridised to the capture probe Comprehensive Exome Panel, with the addition of Twist Universal Blockers and blocking solution for 16 h (all, Twist Bioscience, South San Francisco, CA, USA). The post-capture PCR was performed with xGen Library Amp Primer (0.5 mM, Integrated DNA Technologies, Coralville, IA, USA) for 10 cycles. Quality control was performed with the Quant-iT dsDNA HS assay (Invitrogen, Waltham, MA, USA) and TapeStation HS D1000 assay (Agilent, Santa Clara, CA, USA). Sequencing was performed on NovaSeq 6000 (Illumina, San Diego, CA, USA) using a paired-end 150 nt readout, aiming at 60 M read pairs per sample. Demultiplexing was done using Illumina bcl2fastq v2.20.

### 2.4. Alignment, Variant Calling and Filtering

BALSAMIC v6.0.1 [45] was used to analyse each of the FASTQ files derived from sequencing. In summary, we first quality controlled FASTQ files using FastQC v0.11.5 [46]. Adapter sequences and low-quality bases were trimmed using fastp v0.20.0 [47]. Trimmed reads were mapped to the reference genome hg19 using BWA-MEM v0.7.15 [48]. The resulting SAM files were converted to BAM files and sorted using samtools v1.6 [49,50]. Duplicated reads were marked using Picard tools MarkDuplicates v2.17.0 [51] and promptly quality controlled using CollectHsMetrics, CollectInsertSizeMetrics and CollectAligntmentSummaryMetrics functionalities. Results of the quality-controlled steps were summarised by MultiQC v1.7 [52]. For each sample, small somatic mutations were called using VarDict v2019.06.04 [53], and structural variants were called using Manta v1.3.0 [54]. All variants were finally annotated using Ensembl VEP v100 [55] and vcfanno v0.3.2 [56].

For variant filtering, the following criteria was applied: read depth (DP) > 50, alternative allele depth (AD) > 10, AF > 0.1 (10%) and GNOMAD AF_popmax [57] < 0.005.

In samples with paired normal material, only variants tagged as somatic, likely somatic and strongly somatic by VarDict were kept for downstream analysis. In samples without paired normal material, all variants were considered potentially somatic and thus kept for analysis. For exact parameters used for each of the software, please refer to https://github.com/Clinical-Genomics/BALSAMIC accessed on 20 December 2021 [45].

Further filtering was applied to only studying protein-altering variants. Variants were restricted to transcripts in protein-coding genes, intronic variants and synonymous mutations were filtered out, as were variants with a LOW or MODIFIER (suggesting mutation in the non-coding region) variant impact.

### 2.5. Statistics and Data Analysis

All analyses and plotting were performed in R v 4.1.1. For comparing categorical variables, Fisher’s exact test was used. For comparing continuous variables, a two-tailed *t*-test was used. *p*-values < 0.05 were considered significant.

Enrichment analysis was performed using clusterProfiler [58], with the C2 (curated pathways) collection from MsigDB [59] used as the list of pathways.

## 3. Results

### 3.1. Dataset Summary

After excluding samples of poor quality, primary tumours from 17 patients with recurrence (14 TSCC and 3 BOTSCC), 10 recurrences (i.e., 5 local recurrences and 5 distant metastases) and primary tumours from 18 patients (16 TSCC and 2 BOTSCC) without recurrence remained for further analysis. Adjacent normal material, used to aid somatic variant calling, was available from 13 patients with recurrence and 12 patients without recurrence. For the characteristics of the included patients, please see Table 1.

**Figure 1:**
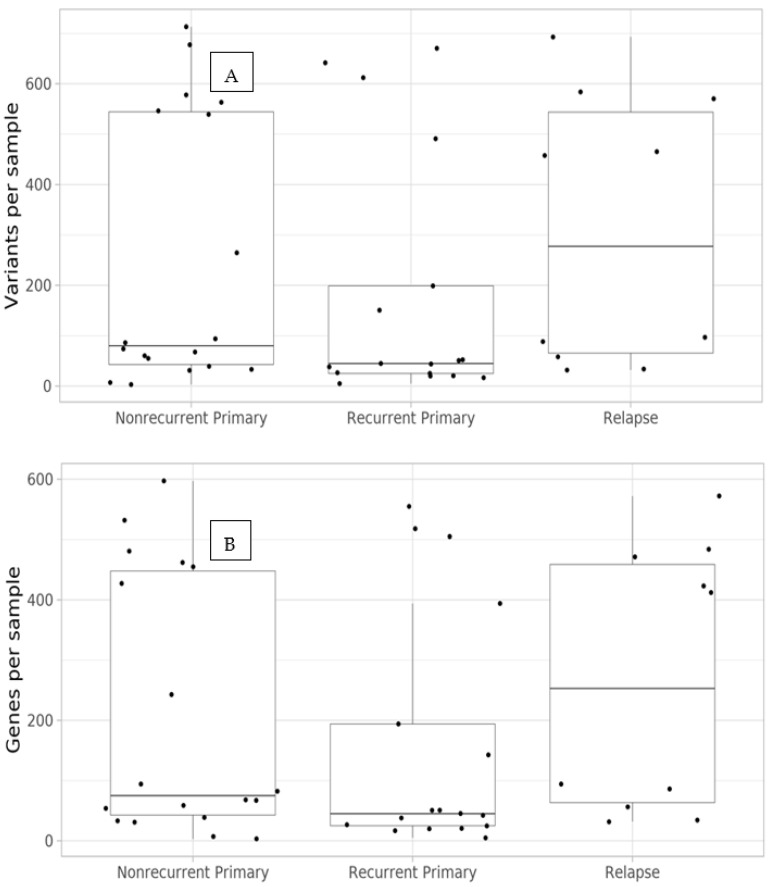
Numbers of identified variants (**A**) and genes impacted (**B**) per sample and cohort. “Nonrecurrent Primary” refers to primary tumours from patients without a local/distant tumour relapse after treatment. “Recurrent Primary” refers to primary tumours from patients with a local/distant relapse after treatment, and “Relapse” refers to corresponding local or distant tumour relapse in these patients.

After variant filtering (see Material and Methods), there were in total 6147 unique variants (SNVs and structural variants) affecting 4184 genes in our dataset. An average of 236 variants affecting 201 genes were identified per sample. The relapses had the highest number of unique variants on average (308 variants in 266 genes), followed by the nonrecurrent primaries (246 variants in 207 genes) and recurrent primaries (183 variants in 156 genes). None of the groups differed significantly from the others in the number of identified variants (Figure 1).

### 3.2. Per-Variant Analyses

We first analysed which specific variants were differentially present between primary tumours from patients with and without recurrences, restricting the analysis to variants present in at least four patients (>20%) of either group while being absent in the other group. The five variants fulfilling these criteria are found in Supplementary Table S1. The only variant enriched (*p* < 0.05) in recurrent samples was a high-impact deletion in the *CDC27* gene, being found in 5/17 primary tumours of patients with recurrence, as well as in one local relapse while being absent from all nonrecurrent patient samples.

Conversely, a substitution in *KCNJ12* was significantly enriched (*p* < 0.05) in the primary tumour samples from patients without recurrence, being present in 5/18 samples. Three more variants in *KRTAP4-11*, *NBPF20* and *LILRB3* were found in four primary tumour samples, each from patients without recurrence, while being absent in all primary tumours of patients with recurrences and the local/distant relapses. However, these did not reach the significance threshold (*p* = 0.1 for all variants).

**Table 1:** Patient and primary tumour characteristics.

Patient Cohort		Patients with Recurrence (%) ^1^	Patients without Recurrence (%) ^1^	Total (%)	*p*-Values
Number of patients		17	18	35	*p*-values
Age		63	63	63	*p* = 0.66
Sex	Male	14 (82%)	13 (72%)	27 (77%)	*p* = 0.7
	Female	3 (18%)	5 (28%)	8 (23%)	
Site	Tonsil Base of tongue	14 (82%) 3 (18%)	16 (89%) 2 (11%)	30 (86%) 5 (14%)	*p* = 0.66
T	T1	1 (6%)	3 (17%)	4 (11%)	T1 + T2 vs.
	T2	7 (41%)	9 (50%)	16 (46%)	T3 + T4
	T3	4 (24%)	5 (27%)	9 (26%)	*p* = 0.3
	T4	5 (29%)	1 (6%)	6 (17%)	
N	N0	1 (6%)	3 (17%)	4 (11%)	N0 + N1 vs.
	N1	1 (6%)	3 (17%)	4 (11%)	N2
	N2	15 (88%)	12 (67%)	27 (77%)	*p* = 0.23
M	M0	17 (100%)	18 (100%)	35 (100%)	*p* = 1
	M1	0 (0%)	0 (0%)	0 (0%)	
TNM Stage	I	0 (3%)	0 (6%)	0 (4%)	TNMI + II vs.
(AJCC 7th Edition)	II III	0 (7%) 2 (25%)	2 (6%) 3 (16%)	2 (8%) 5 (24%)	TNMIII + IV *p* = 0.49
	IV	15 (59%)	13 (65%)	28 (58%)	
TNM Stage (AJCC 8th Edition)	I II III IV	7 (41%) 5 (29%) 5 (29%) 0 (0%)	12 (67%) 5 (28%) 1 (6%) 0 (0%)	19 (54%) 10 (29%) 6 (17%) 0 (0%)	TNMI + II vs. TNMIII + IV *p* = 0.08

^1^ With approximation to full numbers.

Focusing on common variants, 98 variants affecting 80 unique genes (Supplementary Table S2) were present in >25% of all samples independent of outcome (nine or more samples). The most common variants were deletions in *BCLAF1* and *OVCH2*, both present in 12 samples each, followed by a substitution of *OR2T35* found in 11 primary tumour samples.

In 10/17 patients (cases) that had a relapse, samples from either the local or distant relapse were available. These 10 cases of matched local/distant relapses were studied on a per-sample basis in order to find variants that were either: (a) unique to the relapses, suggesting a mutation conferring invasiveness/treatment resistance, (b) unique to primaries, suggesting that these mutations were lost upon clonal expansion after treatment, or (c) occurring in matched primaries and relapses, suggesting mutations in the primary that could predict future relapse.

For (a) and (c), an additional condition was that variants should not be found in any nonrecurrent primary samples either. After limiting the analysis to only variants that were either found in the primary but not in the relapse or in the relapse but not the primary or in nonrecurrent samples; or both in the relapse and the primary but not in nonrecurrent samples in at least 3/10 of these cases, two variants remained (Supplementary Table S3).

**Figure 2:**
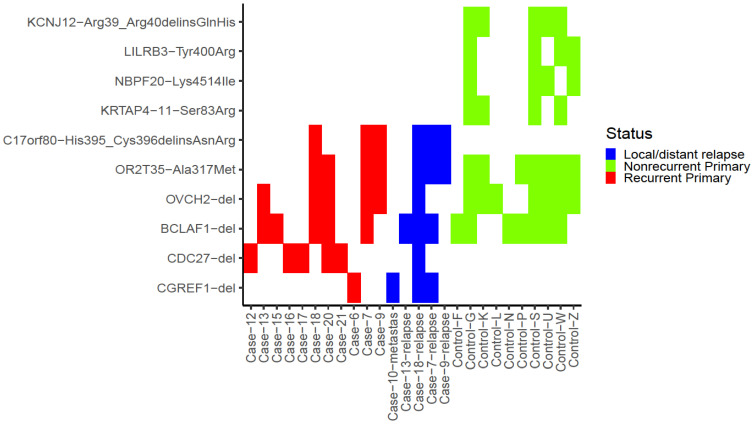
Heatmap of the most commonly occurring variants, variants specific to either primary of patients with local/distant recurrence (denoted as cases) or primaries of nonrecurrent patients (denoted as controls) and variants unique to relapses (denoted metastasis, if distant metastasis, or relapse, if local recurrence). Variants are on the *y*-axis, samples are on the *x*-axis. Colour indicates sample category. Samples where none of the variants were present were excluded from the plot.

A deletion in *CGREF1* was only found in relapses (and in a primary from a recurrent sample without matched relapses), suggesting that this mutation is relapse-specific and may confer invasiveness, and substitution in *C17orf80* was not found in any samples from nonrecurrent patients, suggesting that this specific mutation could be related to relapses, while the other variants are more generally found across many samples regardless of recurrence. The most relevant variants described above are summarised in Figure 2.

### 3.3. Per-Gene Analysis

Subsequently, the same analyses as described above were performed on the gene level. All unique variants per gene were collated, and if any one of these variants was present in a sample, the gene was considered mutated in that sample. No genes were uniquely mutated in any of the tumours of patients with a relapse, while only one gene, *HERC2*, was uniquely mutated in the primaries of 4/18 nonrecurrent patients and in none of the primaries or recurrences of patients with relapses (*p* = 0.1).

**Figure 3:**
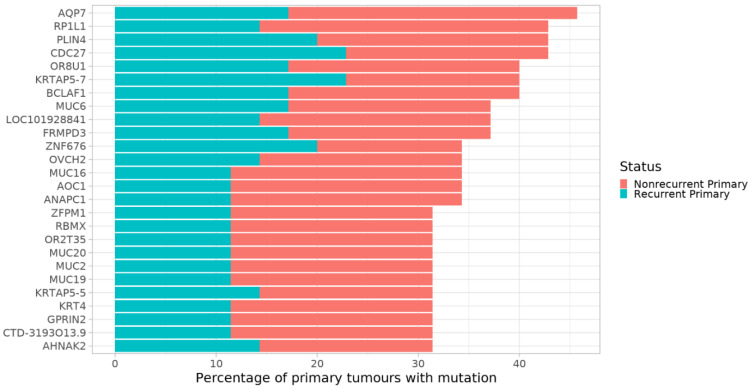
Bar plot of all genes mutated in >30% of primary tumours. Genes are on the *y*-axis. The *x*-axis shows the percentage of all primary tumours where a gene is mutated. Colour indicates recurrent or nonrecurrent status.

A group of 26 genes were mutated in >30% of all primary tumour samples independent of outcome (Figure 3, Supplementary Table S4). The most commonly mutated gene was *AQP7*, while *BCLAF1*, *OVCH2* and *OR2T35*, which had the most common unique variants, were also found among the genes mutated in the largest number of samples.

Interestingly, *CDC27*, where a unique variant was found that was specific for tumours from patients with a recurrence, was also among the most commonly mutated genes. It was mutated in primary tumours of 8/17 patients with recurrence, and in 7/18 primaries of patients without recurrence, as well as in three relapses, with a total of 17 unique variants affecting this gene. Several patients had multiple *CDC27* variants simultaneously, with one of the primary tumours of patients without relapse having 14 unique *CDC27* mutations.

**Figure 4:**
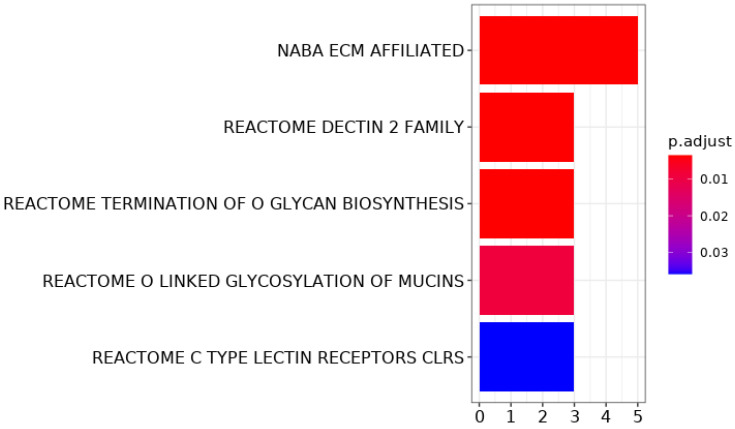
Bar plot of enrichment analysis. Pathways are on the *y*-axis, the number of genes in the pathway overlapping with the 26 most commonly mutated genes on the *x*-axis. The colour shows the *p*-value after the hypergeometric test, adjusted for multiple testing.

Among these 26 genes, there were three keratin-related genes, *KRT4*, *KRTAP5-5* and *KRTAP5-7*, and five different mucins. Upon enrichment analysis, pathways related to the extracellular matrix and carbohydrates were significantly enriched among these 26 commonly mutated genes. The mucins were represented in all these enriched pathways (Figure 4, Supplementary Table S5).

Analysing the relapses as described above, three genes—*CGREF1*, *DCHS2* and *KRT8*—were mutated in three relapses, but no matched primaries. *CGREF1* and *DCHS2* were, however, each additionally mutated in one recurrent primary without matched relapsed samples. *SPATA31D1* and *C17orf80* were mutated in paired primary and relapse from three patients each. *C17orf80* was also mutated in an additional relapse while not being found mutated in the matched primary (Supplementary Table S6).

### 3.4. Mutations in Hotspot Genes

Since genes known to be commonly mutated in HNSCC did not come up among the top genes in our analysis, we specifically analysed two genes known for hotspot mutations in HPV^+^ TSCC/BOTSCC/OPSCC—*FGFR3* and *PIK3CA.* Additionally, we checked for mutations in the TP53 gene to see whether TP53 mutations are enriched among relapses. *FGFR3* was mutated in 2/17 primary tumours of patients with recurrence, and 3/18 patients without recurrence, with one mutation (Ser249Cys) accounting for 4/5 of the mutations and the remaining mutation being adjacent (Arg248Cys). *PIK3CA* was mutated in the primaries of one patient with recurrences and 4/18 patients without recurrence, and additionally in one distant relapse (unmatched to the mutated case). One variant (Glu545Lys) accounted for 4/6 of the mutations. *TP53* was, expectedly in this HPV^+^ cohort, rather rarely mutated. Mutations were only found in two primary tumours of patients with recurrence (Supplementary Table S7).

## 4. Discussion

In this study, WES was performed in laser micro-dissected primary tumours of 18 HPV^+^ TSCC/BOTSCC patients without and 17 patients with recurrence, as well as in 10 corresponding local/distant relapses. A high-impact deletion in the *CDC27* gene was detected only in tumours from patients with a relapse but in none of the tumours from patients without a relapse.

In the entire cohort of primary tumours, we disclosed three variants—deletions in *BCLAF1* and *OVCH2* and substitution in *OR2T35*—and 26 mutated genes that were mutated in >30% of all cases, being part of a global mutational signature of HPV^+^ TSCC/BOTSCC.

The most commonly mutated gene was *AQP7*, mutated in >45% of all primary tumours. Among the most commonly mutated genes were also numerous keratin-associated genes and mucins, and the set of commonly mutated genes was enriched for pathways related to extracellular matrix and carbohydrates.

Moreover, hotspot mutations in *PIK3CA* and *FGFR3* were present in the cohort but were not among the most prominent ones, while mutations in *TP53* tended to be relatively rare, only found in two recurrent patients.

The fact that a deletion in *CDC27* was common (5/17, 29%) in and specific to the primary tumours of patients that relapsed is a novel finding of great interest. With experimental validation, this variant could potentially be used for predicting prognosis and even as a treatment target.

*CDC27* is one of the main components of the anaphase-promoting complex/cyclosome and overexpression and variations in *CDC27* expression may affect the cell cycle, mitosis, cancer pathogenesis and prognosis [60,61]. Thus far, there is no specific targeted therapy for *CDC27*. However, curcumin and miR27a have been suggested to affect *CDC27* function [62,63]. In addition, there are antibodies against *CDC27*, which could be of potential interest to explore in a diagnostic rather than therapeutic setting, as the protein is localised to the nucleus.

Keratin-associated proteins and mucins were enriched among the most commonly mutated genes in our primary cohort, suggesting that these are part of a global HPV^+^ OPSCC mutational signature. *KRTAP5-5* has been linked to motility and invasion [64], while mutations in *KRTAP5-7* have been associated with liver metastases in cancers of unknown primary [65].

Mucins, of which we found five different genes mutated in >30% of primary tumours, have long been associated with cancer, are known to often be overexpressed or structurally altered, interact with the tumour microenvironment and contribute to motility and invasion [66]. Of particular interest for treatment personalisation is the fact that NSCLC with *MUC19* mutations responds very well to anti-PD1 inhibitors [67], making this particular gene interesting to study in the context of checkpoint inhibitor therapy for HPV^+^ OPSCC.

Among the genes affected by the most common unique variants, *BCLAF1* is the best-studied in the cancer setting as an associated transcription factor for Bcl2 [68]. In an experimental system, it has been shown to induce resistance to cisplatin treatment of NSCLC [69], and this variant is also of interest for diagnostic and targeting purposes.

Being the single most commonly mutated gene in our cohort, *AQP7* is also of interest. It encodes a membrane channel with known metabolic roles that is not well studied in the HNSCC setting but has been proposed as a target for breast cancer and is overexpressed in thyroid cancer [70,71,72].

In a study similar to ours, where WES was performed in primary tumours of 51 HPV^+^ OPSCC, of which 35 did not recur, and 16 recurred, and in 33 primaries of HPV^−^ oral cavity cancers and OPSCC, *KMT2D* was found to be the most commonly mutated gene in both primary (14%) and recurrent (42%) HPV^+^ OPSCC [22]. We did detect *KMT2D* mutations in 3/35 (9%) primaries in our cohort, but not at all in the recurrent samples. However, removing our filtering for protein-altering variants, we detected *KMT2D* mutations in 14% of primaries and 20% of relapses, suggesting that differences could be due to more stringent variant filtering in our study. In another similar study, targeted next-generation sequencing using a customised gene panel was performed in 28 HPV^+^ OPSCC and 28 matched HPV^−^ OPSCC [32]. In that study, in the 14 patients with HPV^+^ OPSCC with recurrence, *HRAS*, *PIK3R1*, *STK11* and *TP63* were more frequently mutated in patients with recurrence as compared to those without recurrence [32]. These genes were not very commonly affected in our study, but again highlighting the importance of variant filtering, variants downstream of the *HRAS* gene were seen in 6/35 (17%) of primaries in our cohort but filtered out due to likely not having any consequences at the protein level.

Lastly, an advantage with the present study is that we performed laser micro-dissection of all the tumour material (primary tumours as well as local/distant relapses), ensuring a high tumour yield per sample (>90%). TSCC and BOTSCC are namely characterised by infiltrative growth of tumour nests within a lymphoid stroma and with varying tumour infiltration of lymphoid cells. Therefore, a tumour section or a core biopsy from the tumour tissue block will, even though the tumour area is estimated as above 70%, in most cases give a much lower tumour cell concentration; in many times below 20%, by our experience. By laser microdissection, we were here able to exclude the lymphoid-surrounding tissue, but not tumour infiltrating lymphocytes.

There are obvious limitations in our study, similar to the latter two studies above [22,32], of which one major one is the limited number of patients. The main reason for this is that relapse is uncommon in HPV^+^ OPSCC, which has limited our investigation.

Additional limitations are the use of FFPE samples and the fact that normal tissue was not available in almost one-third of the cases. As all variants called in these cases were counted as somatic, provided they passed filtering for quality and allele frequency, this clearly introduces a risk of false-positive calls. However, our main findings (*CDC27* deletion, commonly affected genes, such as *BCLAF1*) were found both in samples with and without paired normal material. In this case, these samples increase the power of our study while allowing us to confirm the specificity of the findings through their presence in the set of samples with paired normal material. Furthermore, in the cases where normal FFPE tissue was available, one could argue that an optimal approach would be to use peripheral blood as a control in variant calling. However, an advantage of our approach is that normal tissue was, when available, defined by a pathologist and laser micro-dissected from the same tissue block as the tumour. Thus, any paraffin-related artefacts are present in both the control and tumour tissue, avoiding false calling of somatic variants.

## 5. Conclusions

In conclusion, we found a specific *CDC27* variant unique for tumours of HPV^+^ OPSCC patients with relapse, as well a common mutational signature for HPV^+^ OPSCC patients independent of the outcome, comprising keratin-associated proteins and mucins, but also specific variants, such as a *BCLAF1* variant.

These findings may be of potential interest both for predicting prognosis and potential future targeted therapy but do need experimental validation. The possibility to perform WES at reasonable costs suggests targeted sequencing of the genes identified in this study in future studies of HPV^+^ TSCC/BOTSCC in order to evaluate their impact on treatment in a prospective manner, which could ultimately provide additional markers for targeted therapy and treatment planning.

## Data Availability

Please see supplementary data. Due to privacy concerns with WES data, processed, de-identified data will be made available upon reasonable request to the corresponding author.

## Supplementary Materials

The following supporting information can be downloaded at: https://www.mdpi.com/article/10.3390/cancers14010077/s1: Table S1. Variants differing between primaries of recurrent and nonrecurrent patients; Table S2. Variants occurring in >25% of primary tumours; Table S3. Variants associated with relapse; Table S4. Genes mutated in >30% of primary tumours; Table S5. Significantly enriched pathways among commonly mutated genes; Table S6. Genes associated with relapse; Table S7. Hotspot mutations.
